# The potential overdose of heart and left anterior descending coronary artery region during intensity-modulated radiation therapy in patients with esophageal cancer

**DOI:** 10.1093/jrr/rrad100

**Published:** 2023-12-26

**Authors:** Kenji Makita, Yasushi Hamamoto, Hiromitsu Kanzaki, Kei Nagasaki

**Affiliations:** Department of Radiation Oncology, National Hospital Organization Shikoku Cancer Center, Kou-160, Minami-Umenomoto-Machi, Matsuyama, Ehime 791-0280, Japan; Department of Radiology, Ehime University Graduate School of Medicine, 454 Shitsukawa, Toon, Ehime 791-0295, Japan; Department of Radiation Oncology, National Hospital Organization Shikoku Cancer Center, Kou-160, Minami-Umenomoto-Machi, Matsuyama, Ehime 791-0280, Japan; Department of Radiation Oncology, National Hospital Organization Shikoku Cancer Center, Kou-160, Minami-Umenomoto-Machi, Matsuyama, Ehime 791-0280, Japan; Department of Radiation Oncology, National Hospital Organization Shikoku Cancer Center, Kou-160, Minami-Umenomoto-Machi, Matsuyama, Ehime 791-0280, Japan

**Keywords:** left anterior descending coronary artery region, cardiac volume reduction, esophageal cancer, intensity-modulated radiation therapy

## Abstract

This study aimed to investigate the changes in dose distribution in the heart and left anterior descending coronary artery region (LADR) during intensity-modulated radiation therapy (IMRT) in patients with esophageal cancer (EC) treated at our institution. The heart and LADR were delineated on the initial and off-cord boost planning computed tomography (CT) images. Cardiac volume reduction (CVR) was defined as the reduction in cardiac volume between the initial CT and off-cord boost CT at the dose of 36 Gy irradiated. The involved field IMRT plan was created based on each initial and off-cord boost CT image and was analyzed based on the relationship between CVR and heart and LADR dose–volume parameters (Heart-Dmax, Heart-Dmean, Heart-V20, Heart-V30, Heart-V40, LADR-Dmax, LADR-Dmean, LADR-V15 and LADR-V30). Forty patients with EC were investigated between January 2016 and January 2022. The median CVR ratio during radiation therapy (RT) was 5.57% (range, −7.79 to 18.26%). Simple linear regression analysis revealed significant correlations between CVR during RT and changes in the heart and LADR dose–volume parameters. Some patients (>10%) experienced severe changes in the heart and LADR dose distribution. In three cases with reduced heart volume and primary tumor mass, the changes in LADR-V15 and LADR-V30 showed outliers. In conclusion, CVR during RT correlated with an increase in the heart and LADR dose. When both CVR and tumor volume reduction are large, a potential overdose of LADR during RT should be noted in the IMRT era.

## INTRODUCTION

Esophageal cancer (EC) is typically treated with curative-intent concurrent chemoradiotherapy (CCRT) as a definitive treatment option for patients who are either ineligible for or refuse esophagectomy with curative intent [1–3]. Although CCRT is a safe and effective treatment for EC, it may lead to adverse events, such as esophageal, pulmonary and cardiac toxicity [4].

Additionally, cardiac volume reduction (CVR) has been observed during CCRT for EC [5–7]. This phenomenon has been linked to the intravascular volume depletion and dehydration, which are common issues during hospitalization for CCRT treatment [6, 8, 9]. Nevertheless, little attention has been paid to this issue in the past because CVR did not substantially affect the heart dose distribution in the 3D conformal radiation therapy (3D-CRT) era [6].

Recently, cardiac toxicity after chemoradiotherapy for EC has been shown to induce myocardial fibrosis and to lead to worse overall survival [10, 11]. Additionally, in patients with breast or lung cancer, some studies have suggested that the dose to the left anterior descending coronary artery (LAD) correlates with the cardiac events after irradiation [12–14]. Moreover, in the intensity-modulated radiation therapy (IMRT) era, variations in the dose distribution density can lead to elevated doses to the heart and LAD associated with CVR, which did not occur in the 3D-CRT era. Therefore, in our study, we evaluated changes in the heart and LAD region (LADR), which can be accurately contoured without respiratory and cardiac synchronous computed tomography (CT) dose–volume parameters caused by the CVR.

## MATERIALS AND METHODS

Patients with EC who underwent radiotherapy between January 2016 and January 2022 at our institution were retrospectively reviewed. The involved field virtual IMRT plan was created and analyzed to determine the relationship between the CVR and heart and LADR dose–volume parameters. This retrospective study was approved by our institutional review board (RIN 2021-69).

All the patients assessed in this study underwent radiotherapy. Practical 3D-CRT with an elective nodal irradiation (ENI), which targets microscopic mediastinal lymph node metastases that are not evident on imaging, was delivered using 10 MV X-ray from a linear accelerator (Varian Medical Systems, CA, USA) to a total dose of 60 Gy (initial ENI, 40 Gy; off-cord boost irradiation, 20 Gy) with a daily fraction dose of 2 Gy to the EC region. Off-cord boost CT images were obtained at a dose of 36 Gy, and the initial CT and off-cord boost CT images were integrated using the vertebrae. The concurrent chemotherapy with their radiotherapy consisted of cisplatin (70 mg/m^2^ on Days 1 and 29) and 5-fluorouracil (700 mg/m^2^ on Days 1–4 and 29–32) in principle.

### Virtual planning

Virtual IMRT with involved field irradiation (IFI), which targets only visible lesions on imaging studies, was delivered using 6 MV X-ray at a total of 60 Gy in 30 fractions to the EC region. The doses were calculated using the Acuros XB algorithm of our treatment planning systems. These treatment plans were created using the Eclipse planning system (Varian Medical Systems) and 3-mm CT slices obtained during free breathing without cardiac synchronization (HiSpeed NX/I Smart Gantry system; General Electric Healthcare, Little Chalfont, UK). The dose constraints are shown in Supplement 1. The dose constraint of LADR was not used in virtual IMRT planning.

CVR was defined as the reduction in cardiac volume between the initial CT and off-cord boost CT at the dose of 36 Gy irradiated. To evaluate the impact of CVR and LADR movement on the heart and LADR dose–volume parameters, two virtual IMRT plans for each patient with EC were created: (i) using the initial CT images, the heart and LADR were contoured, and IMRT plans with IFI were created (initial IMRT); (ii) using the off-cord boost CT images, the heart and LADR were contoured and were then duplicated to the initial CT images. Subsequently, when CVR occurred, the affected area of CVR was substituted with air density (−1000 Hounsfield units). Finally, using the initial CT images, IMRT plans with IFI were created using the same fields and monitor units as the initial IMRT (boost IMRT).

### Target contouring

The gross tumor volume (GTV) was defined as the primary tumor and metastatic lymph nodes. The primary tumor was identified based on the CT, [^18^F]fluoro-D-glucose positron emission tomography/CT and/or endoscopic findings. The clinical target volume (CTV) for the primary tumor was defined as the tumor volume plus 2–3 cm margins in the craniocaudal direction and 0.5 cm margins in the other directions. The CTV for metastatic lymph nodes was defined as any metastatic lymph node volume plus 0.5 cm margins in all directions. The planning target volume was defined as the overall CTV plus the margins (0.5 cm) in all directions.

### Heart and LADR evaluation

The structure of the whole heart was contoured on both the initial and off-cord boost CT images using the cardiac contouring atlas for radiotherapy [15] with appropriate window settings (window width = 500 Hounsfield units, window level = 50 Hounsfield units). Similarly, the structure of the LADR was contoured on both the initial and off-cord boost CT images using the contouring atlas of the LADR [16]. All contouring procedures were performed by the same radiation oncologist and were reviewed by two other radiation oncologists.

The LADR from the cranial (the level above where the LAD branches from the left main coronary artery) to the caudal (the level of the apex of the heart) was divided equally into two parts, and the LADR movement was measured at the midpoints of the LADR. The LADR movement was quantified by determining (i) the direction of the esophagus (*X*-axis) and (ii) the vertical direction of the *X*-axis (*Y*-axis). The direction to the esophagus was defined as positive on the *X*-axis and the right direction was defined as positive on the *Y*-axis.

The heart and LADR dose–volume histograms (DVH) were generated using a treatment planning system. The percentages of the heart and LADR volumes that received >30 Gy (Heart-V30 and LADR-V30), mean dose (Heart-mean and LADR-mean) and maximum dose (Heart-max and LADR-max) were collected. Additionally, the percentage of the heart volume that received >20 Gy (Heart-V20) and >40 Gy (Heart-V40) and the percentage of LADR volume that received >15 Gy (LADR-V15) were collected. A change of ≤10, >5 and >10% in cardiac DVH parameters was defined as mild and severe changes, respectively.

### Statistical analysis

Statistical analyses were conducted using JMP software (version 14.3.0; SAS Institute, Cary, NC, USA) and EZR (Saitama Medical Center, Jichi Medical University, Saitama, Japan), which is a graphical user interface for R (The R Foundation for Statistical Computing, Vienna, Austria, version 3.5.0) [17]. Extreme values (outliers) were identified using the Smirnov-Grubbs analysis. Simple linear regression was applied to evaluate the correlation between the CVR and LADR dose changes. Data are presented as regression coefficients with 95% confidence intervals and *P*-values. In addition, multiple linear regression analysis was conducted to investigate the correlation between LADR movement and LADR dose change. Fisher’s exact test were used to assess the significance of group differences in the variables. A *P*-value of <0.05 was considered to be statistically significant.

## RESULTS

Between January 2016 and January 2022, 143 patients received definitive radiation therapy (RT) with or without concurrent chemotherapy at our institution. Of these, patients with the following characteristics were excluded from the study: (i) surgical therapy, including endoscopic treatment before RT (*n* = 9); (ii) absence of replan CT image (*n* = 13); (iii) incomplete RT (*n* = 2); (iv) non-use of the daily fraction dose of 2 Gy (*n* = 23); (v) double cancer diagnosis (*n* = 16); (vi) presence of distant metastases (*n* = 17); (vii) primary tumor located in the upper thoracic esophagus (*n* = 12) and (viii) incongruent between the cranial and caudal points on the initial CT and off-cord boost CT images by >5 mm (*n* = 11). Finally, a retrospective analysis was performed on the remaining 40 patients with EC (male/female = 34/6; median [range], 68 [48–88] years) who received definitive RT. Details of the patient characteristics are shown in Table 1.

**Table 1:** Patients’ characteristics

Characteristic	No. of patients	%
Age		
<70 years	22	55.0
≥70 years	18	45.0
Sex		
Male	34	85.0
Female	6	15.0
PS		
0	17	42.5
1	23	57.5
BMI		
<23	22	55.0
≥23	18	45.0
Clinical stage (UICC eighth)		
1	13	32.5
2	15	37.5
3	11	27.5
4	1	2.5
T stage (UICC eighth)		
1	11	27.5
2	13	32.5
3	13	32.5
4	3	7.5
Location		
Mid-thoracic	19	47.5
Low thoracic	6	15.0
Mid-low thoracic	15	37.5
Smoking		
Yes	28	70.0
No	7	17.5
Unknown	5	12.5
Hypertension		
Yes	22	55.0
No	18	45.0
Hypercholesterolemia		
Yes	3	7.5
No	37	92.5
Chemotherapy		
Yes	37	92.5
Low dose	10	25.0
Standard dose	27	67.5
No	3	7.5

PS = performance status, BMI = body mass index, UICC = Union for International Cancer Control.

**Fig. 1:**
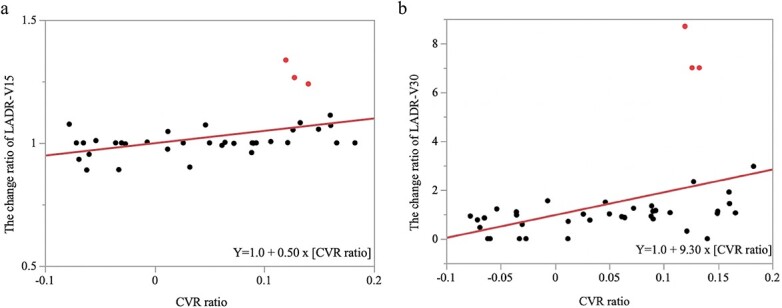
(**a**) Simple linear regression analysis between the change ratio of LADR-V15 and CVR ratio. The red point shows three outliers (24.0, 26.5 and 33.7% increase) for LADR-V15 in the Smirnov-Grubbs analysis. V15 = volume receiving 15 Gy. (**b**) Simple linear regression analysis between the change ratio of LADR-V30 and CVR ratio. The red point shows three outliers (700.0, 700.0 and 975.0% increase) for LADR-V30 in the Smirnov-Grubbs analysis. V30 = volume receiving 30 Gy.

**Fig. 2:**
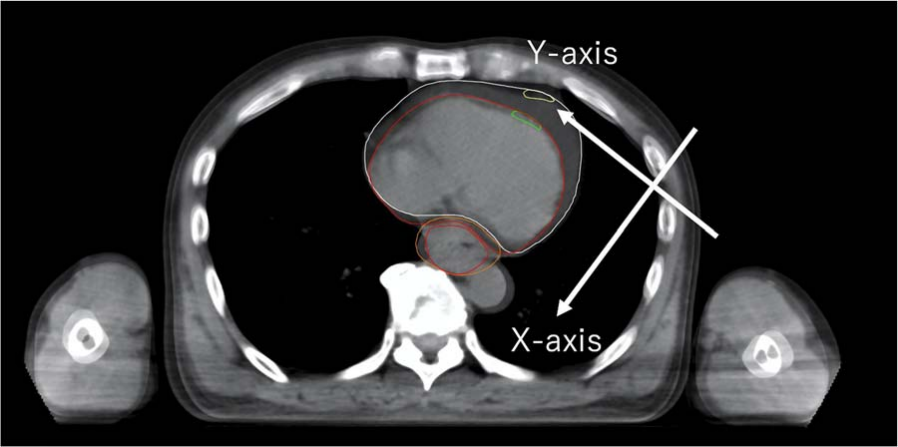
A case of severe change of LADR-V15 and LADR-V30. This case had severe CVR (>10%) and reduction of primary tumor mass (orange line to red line of primary tumor mass). Large LADR movement was observed (yellow line to green line of LADR). Vxx = volume receiving xx Gy.

The median CVR ratio during RT was 5.57% (range, −7.79 to 18.26%). Among the 40 EC patients, 11 patients (27.5%) experienced severe CVR (>10%) and 10 patients (25.0%) experienced mild CVR (≤10%, >5%). Furthermore, simple linear regression analysis showed that changes in all heart and LADR dose distributions were significantly correlated with the CVR (Table 2). Severe changes (>10%) in Heart-max, Heart-mean, Heart-V20, Heart-V30 and Heart-V40 were 1 (2.5%), 7 (17.5%), 7 (17.5%), 13 (32.5%) and 19 (47.5%) patients, respectively. Severe changes (>10%) in LADR-max, LADR-mean, LADR-V15 and LADR-V30 were 4 (10.0%), 5 (12.5%), 4 (10.0%) and 14 (35.0%) patients, respectively. In the Smirnov-Grubbs analysis, three outliers (24.0, 26.5 and 33.7% increase) for LADR-V15 were found in the change in LADR dose distribution (Table 3, Fig. 1a). Similarly, three outliers (700.0, 700.0 and 975.0% increase) for LADR-V30 were found in the change in LADR dose distribution (Table 3, Fig. 1b). These outliers were found in patients with severe CVR (>10%) and a reduction in primary tumor mass. A case of severe change in LADR-V15 and LADR-V30 is shown in Fig. 2. There was no significant difference between the T stage and the incidence of mild or severe CVR (mild CVR, *P* = 0.92; severe CVR, *P* = 0.60, respectively). Furthermore, there was no significant difference between the T stage and the incidence of severe change of heart dose or LADR dose (Heart-max, *P* = 0.51; Heart-mean, *P* = 0.11; Heart-V20, *P* = 0.11; Heart-V30, *P* = 0.63; Heart-V40, *P* = 0.75; LADR-max, *P* = 0.78; LADR-mean, *P* = 0.26; LADR-V15, *P* = 0.26; LADR-V30, *P* = 0.62, respectively).

**Table 2:** Simple linear regression analysis for the factors correlated with heart and LADR dose change according to CVR during radiotherapy treatment

Variables	Initial DVH	Off-cord boost DVH	Coefficient	95% CI	*P*-value
Heart-max (Gy)	69.5 (63.7–82.1)	69.9 (63.0–81.9)	0.062	0.027–0.097	0.001
Heart-mean (Gy)	27.6 (12.7–38.9)	29.1 (13.2–43.6)	0.418	0.249–0.586	<0.001
Heart-V20 (%)	70.9 (26.4–95.3)	74.6 (27.4–98.8)	0.410	0.222–0.598	<0.001
Heart-V30 (%)	33.8 (11.5–74.4)	38.7 (12.1–85.6)	0.796	0.504–1.088	<0.001
Heart-V40 (%)	15.5 (4.7–43.6)	16.8 (5.9–56.5)	0.859	0.369–1.350	0.001
LADR-max (Gy)	36.8 (26.0–64.7)	37.0 (25.6–65.7)	0.577	0.357–0.797	<0.001
LADR-mean (Gy)	21.1 (8.6–33.5)	21.9 (7.7–36.6)	0.495	0.271–0.719	<0.001
LADR-V15 (%)	94.2 (27.2–100.0)	95.2 (24.2–100.0)	0.409	0.069–0.749	0.020
LADR-V30 (%)	6.1 (0.0–92.0)	6.7 (0.0–97.2)	0.403	0.367–1.649	0.003

Initial and off-cord boost DVH parameters; median (range).

**Table 3:** Three outliers for change ratio of LADR-V15

Patient	Age	Sex	PS	Tumor location	T stage	Chemotherapy	LADR-V15/V30 of initial CT (%)	LADR-V15/V30 of off-cord boost CT (%)	Change ratio of LADR-V15/V30 (%)	Change ratio of CVR (%)
1	73	Man	1	Ut-Mt	3	FP	39.2/0.3	48.6/2.1	24.0/700.0	14.0
2	66	Man	1	Mt	3	FP	60.3/0.4	80.6/3.9	33.7/975.0	12.0
3	58	Man	1	Ut-Mt	4	FP	53.3/0.3	67.7/2.1	26.5/700.0	12.7

Ut = upper thoracic esophagus, Mt = middle thoracic esophagus, FP = 5-fluorouracil and cisplatin.

The median LADR movement during RT was 0.47 cm (range, 0.00–2.02 cm) on *X*-axis and 0.10 cm (range, −0.74 to 1.73 cm) on *Y*-axis. Severe changes (>1 cm) in LADR movement along the *X*- and *Y*-axes were observed in nine (22.5%) and two patients (5.0%), respectively. On multiple linear regression analysis, these LADR movements (*X*- and *Y*-axes) correlated with CVR during RT (Supplement 2). Moreover, a simple linear regression analysis showed that LADR movement along the *X*-axis affected the change in LADR dose distribution (Supplement 3).

## DISCUSSION

Our study demonstrated a significant correlation between CVR during RT and the changes in heart and LADR dose–volume parameters. However, the majority of cases with CVR during RT did not have a large impact on the heart and LADR dose increases. Some cases had severe (>10%) heart and LADR dose increases, and in cases with a reduction in primary tumor mass and severe CVR, the change in LADR dose distribution showed outliers, which led to a remarkable increase in the LADR dose, which could not be predicted from only the CVR rate.

Many studies showed that RT to the heart can cause cardiac impairment, leading to a decline in cardiac function [6, 7, 10–14, 18–20]. Furthermore, cardiac damage can occur even in the early stages of CCRT for patients with EC and the heart volume receiving a high dose may influence the extent of cardiac damage [7, 19]. Therefore, optimization of the cardiac dose distribution is important for improving the treatment outcomes. Although CVR during RT may increase the risk of a higher dose to the heart compared with the treatment plan, our study found that, although some cases experienced the severe (>10%) increase of heart dose, in the majority of cases, the influence of CVR during RT to heart dose distribution was not large in the IMRT era as well as in the 3D-CRT era [6]. This suggests that most patients experiencing CVR during RT can be treated safely while adhering to the dose constraints in the treatment plan.

Furthermore, the LAD dose may be associated with late cardiac adverse events [12–14]. CVR during RT has the potential risk of affecting the LAD dose distribution in IMRT, which is a more precise radiation method than the 3D-CRT. In our study, the influence of CVR during RT to the LADR dose distribution was not large in the IMRT era. However, some patients experienced a severe (>10%) increase in the LADR dose. Furthermore, three cases with outliers (remarkable increase in LADR-V15 and LADR-V30) showed a comparatively large reduction in both the heart and tumor volumes. A large LADR dose increase owing to a large heart and tumor volume reduction should be noted.

In patients with non-small cell lung cancer, an increase in LAD-V15 was found to be a significantly unfavorable factor for major adverse cardiac events and to correlate with unfavorable prognosis [12, 21]. In addition, an increased LAD-V30 in EC patients displayed a significant positive correlation with major adverse cardiac events [14]. Therefore, CVR-driven LADR-V15 and LADR-V30 increases may be unfavorable for EC patients treated with RT. Although the cut-off values for LAD-V15 and LAD-V30 were both 10% in these studies, the 10% cut-off value of LADR-V15 renders it difficult to adhere to the dose constraints in patients with mid-to-lower thoracic EC. The three LADR-V15 outliers in our study displayed >10% LADR-V15 on initial DVH. Conversely, these three outliers exhibited very large LADR-V30 change ratios, but their LADR-V30 values on the initial DVH were low enough. It was thus not possible to assess the clinical impact of LADR-V15 and LADR-V30 changes due to CVR in our study. To date, the ideal cut-off value for these parameters has been controversial due to the limited number of studies. Therefore, in IMRT planning, increasing the intensity of the oblique entry beam from the front right to the back left should be considered as a means of reducing the LADR prescribed dose because the LADR is the path of the oblique entry beam from the front left to back right. Despite the unresolved aspects of the relationship between LAD dose distribution and adverse cardiac events in patients with EC, it is important to reduce LADR-V15 and LADR-V30 in initial DVH as much as possible and to consider the changes in LADR dose distribution in response to CVR and tumor volume reduction.

Our study has some limitations. First, the sample size was small, which may have contributed to outlier identification. Second, because of the limited CT image quality owing to free breathing, lack of cardiac synchronization and absence of intravenous contrast medium administration, the LAD was not adequately evaluated. Therefore, we contoured the LADR as accurately as possible and evaluated its movement between the initial and off-cord boost-plan CT. In our study, the variation in LADR due to free breathing, without cardiac synchronization and without intravenous contrast medium administration was judged by three observers (radiation oncologists) to have an acceptable impact on contouring. However, the variations in the right coronary artery (RCA) and RCA region were too large to have an acceptable impact on contouring. Therefore, further prospective studies are required to evaluate the changes in the LAD and RCA dose distributions. Third, the tumor shrinkage rate could not be evaluated accurately because GTV was based on a clip implanted by endoscope only before the initial CT simulation. Therefore, the influence of tumor shrinkage could not be assessed. Additionally, although T stage did not correlate with DVH parameters, tumor shrinkage may affect the DVH parameters because cases with severe CVR and mass shrinkage in off-cord boost CT image showed the outliers of LADR-V15 and LADR-V30 when the primary tumor formed mass. Finally, DVH parameters at a dose of 60 Gy irradiated could not be evaluated because of the lack of CT images. However, this study suggested that replanning is important to reduce the risk of unexpected LADR dose increases in cases with large CVR and tumor shrinkage at the dose of 36 Gy irradiated. Despite these limitations, our study showed an important finding in daily clinical practice, especially in IMRT planning, because there have been few reports on CVR during RT for patients with EC and no reports on the changes in LADR and heart dose distribution due to CVR in IMRT plans.

## CONCLUSION

In this study, CVR during RT was correlated with an increase in the heart and LADR doses, but the influence was not substantial. However, some cases showed a severe (>10%) increase in the heart and LADR dose distributions, and a large reduction in the heart and tumor volumes resulted in a substantial increase in the LADR dose. Although further studies are required, the occurrence of a large LADR dose increase during IMRT in patients with EC should be considered when assessing adverse cardiac toxicity.

## CONFLICT OF INTEREST

The authors declare that they have no conflict of interest.

## FUNDING

The authors have no funding for this research.

## Supplementary Material

Supplement_1_rrad100

Supplement_2_rrad100

Supplement_3_rrad100
