# Supplementary material for: The potential overdose of heart and left anterior descending coronary artery region during intensity-modulated radiation therapy in patients with esophageal cancer
Source: J Radiat Res. 2023 Dec 26;65(2):238–43. doi: 10.1093/jrr/rrad100 (PMC10959431; doi:10.1093/jrr/rrad100)
Supplement: Supplement_1_rrad100 [file supplement_1_rrad100.docx]

**Supplement 1.** Dose constrain for virtual IMRT plan

| structure | index | objectives | acceptable |
| --- | --- | --- | --- |
| PTV | D98% | >51 Gy | >45Gy |
|  | D95% | 60 Gy | >57 Gy |
|  | D2% | <69 Gy | <72 Gy |
| Body | max | <72 Gy | <75 Gy |
| PRV_Spinal Cord | max | <45 Gy | <50 Gy |
| Lung | V20 | <30% | <35% |
|  | V5 | <60% | <65% |
|  | mean | <17 Gy | <20 Gy |
| Heart | V30 | <35% | <45% |
|  | mean | <20 Gy | <26 Gy |
| Esophagus | max | <75 Gy | <80 Gy |
|  | V70 | <15% | <20% |
|  | V50 | <35% | <40% |
|  | mean | <34 Gy | <40 Gy |
| Liver | V30 | <30% | <40% |
|  | mean | <20 Gy | <30 Gy |
| Kidneys | V20 | <25% | <32% |
| PRV_Stomach/Bowel | max | <50 Gy | <54 Gy |

Dxx%, dose to xx% of the organ; VxxGy, volume receiving xx Gy

PRV, planning organ at risk volume; PRV_Spinal Cord, Spinal Cord plus 3mm margin; PRV_Stomach/Bowel, Stomach/Bowel plus 5mm margin
