# Supplementary material for: The potential overdose of heart and left anterior descending coronary artery region during intensity-modulated radiation therapy in patients with esophageal cancer
Source: J Radiat Res. 2023 Dec 26;65(2):238–43. doi: 10.1093/jrr/rrad100 (PMC10959431; doi:10.1093/jrr/rrad100)
Supplement: Supplement_2_rrad100 [file supplement_2_rrad100.docx]

**Supplement 2.** Univariate and multivariate analyses for the factors correlated with LADR movement according to CVR during radiotherapy

| variables | Univariate analysis | | |  | Multivariate analysis | | |
| --- | --- | --- | --- | --- | --- | --- | --- |
|  | coefficients | p-value | 95%CI |  | coefficients | p-value | 95%CI |
| LADR movement (X-axis) | 0.104 | <0.001 | 0.068-0.140 |  | 0.083 | <0.001 | 0.044-0.122 |
| LADR movement (Y-axis) | 0.093 | <0.001 | 0.047-0.139 |  | 0.047 | 0.033 | 0.004-0.091 |

CVR, cardiac volume reduction; LADR, left anterior descending artery region
