# Supplementary material for: The potential overdose of heart and left anterior descending coronary artery region during intensity-modulated radiation therapy in patients with esophageal cancer
Source: J Radiat Res. 2023 Dec 26;65(2):238–43. doi: 10.1093/jrr/rrad100 (PMC10959431; doi:10.1093/jrr/rrad100)
Supplement: Supplement_3_rrad100 [file supplement_3_rrad100.docx]

**Supplement 3.** Simple linear regression analysis for the factors correlated with LADR dose change according to LADR movement during radiotherapy

|  | variables | coefficient | 95%CI | p-value |
| --- | --- | --- | --- | --- |
| X-axis | LADR-max | 5.263 | 3.514-7.051 | <0.001 |
|  | LADR-mean | 4.814 | 2.755-6.872 | <0.001 |
|  | LADR-V15 | 2.634 | 0.870-4.398 | 0.005 |
|  | LADR-V30 | 6.031 | 2.962-9.101 | <0.001 |
| Y-axis | LADR-max | 3.088 | 1.117-5.058 | 0.003 |
|  | LADR-mean | 2.433 | 0.252-4.613 | 0.030 |
|  | LADR-V15 | 2.261 | -0.936-5.459 | 0.160 |
|  | LADR-V30 | 0.848 | -3.225-4.921 | 0.676 |

LADR, left anterior descending artery region; Vxx, volume receiving xx Gy
